# The nature and fate of natural resins in the geosphere. XII. Investigation of C-ring aromatic diterpenoids in Raritan amber by pyrolysis-GC-matrix isolation FTIR-MS

**DOI:** 10.1186/1467-4866-7-2

**Published:** 2006-03-01

**Authors:** Ken B Anderson

**Affiliations:** 1 Department of Geology, Southern Illinois University Carbondale, Carbondale, IL, 62901, USA

## Abstract

Upper Cretaceous amber from the Raritan Formation (Sayerville, New Jersey) has been investigated by Pyrolysis-GC-MS and Pyrolysis-GC-matrix isolation FTIR-MS. Results establish the existence of two distinct forms of amber in this deposit. Both forms are Class Ib ambers, but they are unambiguously differentiated on the basis of their (intact) diterpenoid composition. The presence of callitrisate in both forms, and cupraene in samples designated form 1, strongly suggest that both derive from related-but-distinct species within the Cupressaceae.

In addition to callitrisate, dehydroabietate and analogous 17-nor-, 16,17-dinor- and 15,16,17-trinor- analogues of these compounds are also observed. The distributions of these products in multiple samples suggest that they are the result of biological emplacement, rather than diagenetic modification of the parent compounds. This indicates that the distributions of diterpenes observed in these samples are representative of the original bioterpenoids and, hence, are useful for chemotaxonomic analyses.

## Introduction

Amber, the fossil form of plant resins, occurs in terrestrial sediments throughout the geologic record from about the early-middle Mesozoic forward and is relatively common in Cenozoic sediments [1]. Older fossil "resins" are known from the occurrence of resinites in coals as old as the Carboniferous, but there is emerging evidence that these materials may be a distinct group of products not necessarily directly related to the terpenoid-based resins common in modern plants and recognized in the sedimentary record as amber [2-4].

Geologic classification/nomenclature of ambers is of little value. Ambers from separate deposits are known by a plethora of names which have no relationship to structural properties or composition. Even within a single deposit, ambers of different clarity, color, friability and/or other properties may be referred to by discrete names, which can result in serious confusion and misclassification. Geochemically, ambers are classified on the basis of their structural properties into groups with comparable structural characteristics [1,5-7]. Class I ambers, those based primarily on polymers of labdanoid diterpenes (I), appear to be by far the most common form of amber in the geosphere. These resins appear to have evolved by the Early Mesozoic (Triassic) [Anderson, unpublished results], and diversified through a wide range of species. In modern plants, resins of this basic composition occur in numerous families including both Angiosperms and Gymnosperms.

Note: Chemical structures referred to in the text are illustrated in Appendix 1. Structures are identified by parenthetical Roman Numerals, with specific derivatives identified where appropriate by a lower case alphabetic designation.

Class I ambers are further differentiated into Class Ia, Ib and Ic on the basis of details of the nature of the labdanoid diterpenes on which their macromolecular structure is based, and on the presence or absence of succinic acid [1,5-7]. Class Ia and Ib ambers are based on polymers of labdanoid diterpenes with "*regular*" stereochemistry. That is, structures including and related to communic acid, communol etc (II). Class Ia and Ib ambers are differentiated in that Class Ia ambers incorporate significant amounts of succinic acid into their macromolecular structure, presumably as a natural cross-linking agent (e.g. Baltic amber, a.k.a. succinite) whereas this product is absent in Class Ib ambers. Class Ic ambers are based on a macromolecular structure derived primarily from labdanoids with a (so called) "*enantio*" configuration, such as, for example, ozic acid, ozol and related hydrocarbons (III). The well known ambers found in Central America, especially those mined in the Dominican Republic and Mexico that are believed to be derived largely or exclusively from *Hymenaea *[8] are of this type. In all cases, in addition to the overall macromolecular network, Class I ambers may include other occluded (or polymer bound) compounds. While these are not usually structurally significant overall, these may be of considerable interest for their chemotaxonomic value.

Numerous sizable amber deposits are known around the world. Ambers are particularly well known in Europe, especially the significant deposits located in the Baltic region where ambers have been collected and/or mined for millennia and are deeply embedded into regional history and folklore [9]. New world deposits are also known and significant amounts of amber are now mined and exported from the Dominican Republic and Mexico. Numerous smaller deposits are also known. In most cases these are not prospected commercially, at least not on a large scale, but they represent a potentially important geologic and paleontological record and can be of significant scientific interest.

Scientific interest in ambers is largely related to either investigation of the amber itself or to investigation of materials (especially insects and other small organisms) that are preserved within it. Fossiliferous material within amber is often preserved to a higher degree of fidelity than perhaps any other medium. Grimaldi [10] has even coined the term "ambalmed" to describe this type of preservation. There is also increasing interest in the use of ambers as chemotaxonomic indicators. This latter area of interest derives from that fact that the compositions of many modern resins are, at least in some degree, characteristic of the plants from which the resins are produced [1,11] and to the extent that compositional characteristics are preserved in the fossil resins, detailed analysis of ambers may serve as a useful means for investigation of paleovegetation, and hence, paleoenvironmental conditions. As also noted by others [11,12] this approach is necessarily complicated by potential diagenetic alteration of resin components, by the unknown degree of evolutionary change in resin composition since biosynthesis of the original resin from which the amber was formed, and by the limits of analogy between the composition of modern resins and their fossiliferous analogues. Nevertheless, numerous authors have pursued and/or are now pursuing this approach [8,13-16] and it is clear that despite these potentially complicating factors, useful data can be generated.

Amber is found in numerous locales in North America, including several locations on the Atlantic Coastal Plain. Perhaps the largest and best known of these (Atlantic Coastal Plain) deposits occurs in the Raritan formation, which extends from Staten Island across parts of New Jersey. Amber from this area was first reported in 1821 [17] and since then there have been numerous reports of investigations of these deposits, most of which have been recently reviewed by Grimaldi et al. [18,19] It is now well established that these deposits are Upper Cretaceous (Cenomanian-Turonian). Amber from these deposits is usually found in clays, often in association with woody remains.

Langenheim and Beck [13]and subsequently Grimaldi et al [18] concluded on the basis of infrared and Py-MS analyses that these ambers were likely of *Araucariaceous *origin. Grimaldi, Wampler and Shedrinski [19] subsequently revised this conclusion and suggested a *Pinaceous *(or *Taxodiaceous*) origin, based on the results of pyrolysis-gas chromatography (Py-GC) and pyrolysis-gas chromatography-mass spectrometry (Py-GC-MS) analyses. However, the high pyrolysis temperatures (650°C, 15 sec) used for these analyses tends to favor the formation of secondary products [21].- hence the high proportion of low molecular weight aromatic products identified by these authors. (Note also, many of the compounds identified in that report cannot be assigned with confidence on the basis of comparison of MS results with library spectra due to the existence of isomeric compounds with essentially identical mass spectra, e.g. [20].) Furthermore, the absence of any provision for derivatization of acidic structures severely hampers chromatographic performance in analyses of these types of samples and results in extreme bias in the results obtained [21]. As a result, the Py-GC and Py-GC-MS data reported by these authors is of limited chemotaxonomic value.

In the present study, samples of Raritan amber have been analyzed by Py-GC-MS and Py-GC-matrix isolation FTIR-MS (Py-GC-mi FTIR-MS) with in situ methylation of acidic products by tetramethyl ammonium hydroxide (TMAH). The observed distribution of products is discussed both with respect to chemotaxonomic significance and also in relation to potential diagenetic modification of the original diterpene distributions.

## Experimental

**Figure 1:**
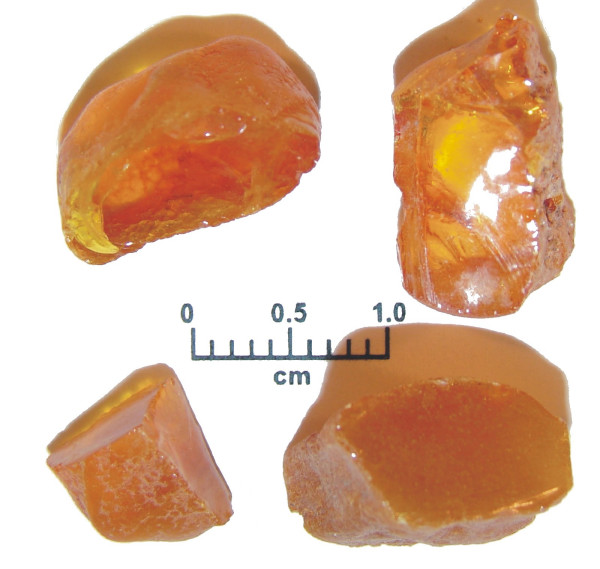
Raritan amber.

Samples were collected from the Raritan formation (Upper Cretaceous, Cenomanian-Turonian), Sayerville, New Jersey, by Mr. Allen Graffham and donated for analysis. Samples were water-washed and unpolished. A total of five individual blebs were used for these analyses. All were visually comparable, being generally clear, yellow-orange in color and irregular but rounded in shape and ranging in size from ~0.7 cm to 2 cm (Figure 1). Each bleb was shattered and appropriately sized individual fragments randomly selected for analysis. Each sample was subjected to a minimum of 10 analyses to ensure that data obtained are representative.

Py-GC-MS analyses were carried out as previously described [21], except that a newer model CDS 2500 pyrolyzer was used. All data reported were obtained by pyrolysis (10 sec at 480°C) in the presence of TMAH to ensure methylation of carboxylic acids liberated by pyrolysis. Separation and analysis of pyrolysate components was accomplished using an Agilent 6890 GC system coupled with an Agilent 5973 MSD and a ClearIR matrix isolation FTIR system. Separation was achieved using a 60 m DB 1701 (0.25 mm, 0.25 μm film thickness), capillary column with some additional analyses completed using a 30 m VB-5 (0.25 mm, 0.25 μm film thickness) as necessary. Carrier gas (He or 2% Ar in He for mi FTIR analyses) was held at 1 ml/minute. Temperature programming was as follows: T_initial _= 40°C (4 minutes); ramp = 4°C/min to T_final _= 280°C. Temperature was then held isothermally for at least 16 minutes.

## Results and discussion

### Pyrolysis-GC-MS

Five discrete samples from this deposit were analyzed by Py-GC-MS. The resulting data clearly indicate the existence of two related-but-distinct forms of amber. These two materials are visually indistinguishable, but are clearly differentiable on the basis of the compounds observed in their pyrolysates. This finding contradicts the earlier finding that ambers from this site are chemically uniform [19].

**Figure 2:**
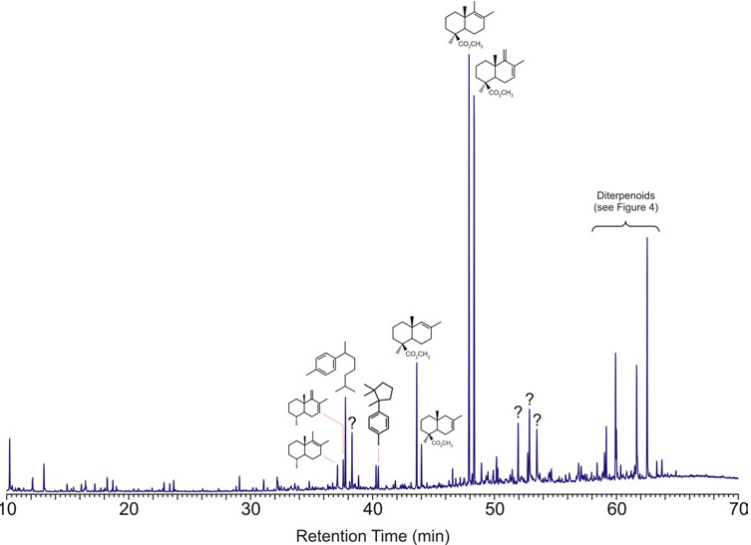
Total ion chromatogram obtained from Raritan amber form 1 by pyrolysis at 480°C in the presence of TMAH (60 m DB-1701 column). Additional details of the distribution of intact diterpenoids are discussed below and given in Figures 4 and 6.

The total ion chromatogram (TIC) obtained from Raritan amber form 1 is illustrated in Figure 2. The pyrolysate of this form is dominated by characteristic bicyclic products typical of Class Ia and Ib ambers (IV-VII). Succinic acid (dimethyl succinate) is absent in these samples. Therefore, these samples are identified as typical Class Ib ambers [5-7].

Polymer-derived bicyclic products observed are dominated almost entirely by carboxylic acids derived from communic acid (IVa-VIIa). A-ring defunctionalized analogues (IVe/f-VIIe/f) of these characteristic bicyclic acids [7] are also present. Communol (IIc) and Biformene (IId) derived characteristic bicyclic products (IVb/c-VIIb/c) are absent or present in only trace amounts, although small amounts of methyl ether products (IVd-VIId) are observed in some samples. Monoterpenoids are present in some samples in low, variable amounts. Cuparene (VIII) is observed as a minor component in the pyrolysate of this form.

**Figure 3:**
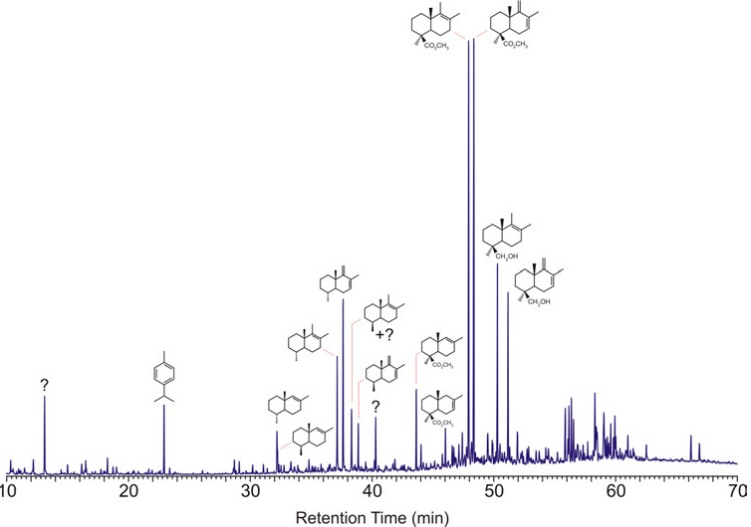
Total ion chromatogram obtained from Raritan amber form 2 by pyrolysis at 480°C in the presence of TMAH (60 m DB-1701 column).

Data for the second form are also indicative a Class Ib amber, and give comparable C_14_/C_15 _ (IV+V)/(VI+VII) ratios for the characteristic polymer-derived bicyclic products, consistent with these samples being of comparable maturity [5] (as expected for co-deposited materials). However, these samples show an entirely different pattern of non-polymer-derived diterpenoids from that which is observed for samples of form 1 and show evidence (IVb-VIIb) for incorporation of significant amounts of communol (IIc) into their macromolecular structure. These samples have been designated as Raritan form 2. TIC data for this form are illustrated in Figure 3.

**Figure 4:**
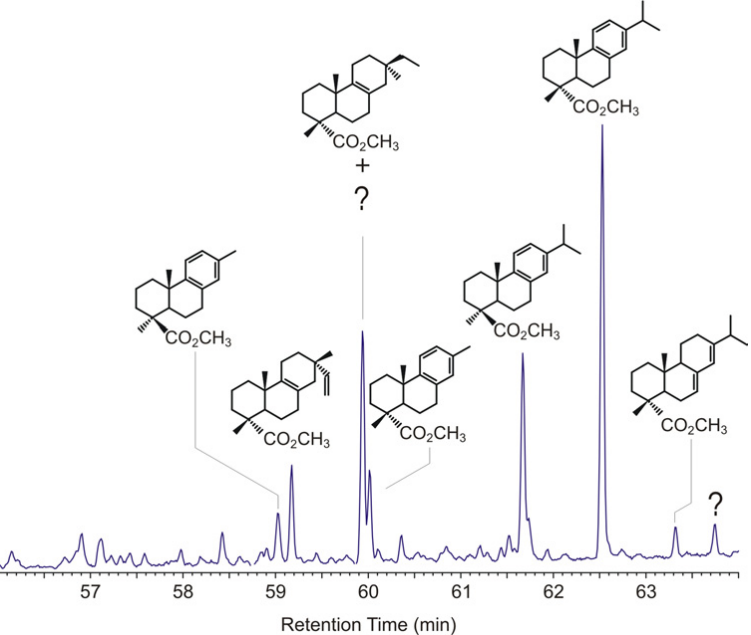
Distribution of major intact diterpenoids observed in the pyrolysate of Raritan form 1. Assignments indicated are based on comparison of both MS data and FTIR data with literature data and data from authentic standards.

The distribution of the major intact diterpenoids observed in the pyrolysates of Raritan form 1 amber is illustrated in Figure 4. (This figure is an enlargement of the data illustrated in Figure 2 so that details are more readily apparent.) Supporting supplemental data are also given in Additional File 1. (Supplemental data are also available on line at http://suplimental data Figure 4) Unbound diterpenoids present in these samples are dominated by methyl dehydroabietate (IXb) and methyl callitrisate (Xb), together with lesser amounts of pimarates (XI), isopimarates (XII), abietate (XIII) and several dinor-diterpenoids structurally related to dehydroabietate and callitrisate. As is apparent from the data illustrated in Figure 2, the distribution of diterpenoids observed in form 2 is considerably more complex than that which is observed in form 1. Effort aimed at identification of many of these products is ongoing, and will be reported separately. However, the pyrolysate of this material includes many of the same C-ring aromatic diterpenes observed in the pyrolysate of form 1.

### Chemotaxonomy and paleobotany

Grimaldi et al[19] conclude that Raritan ambers may be derived from a Pinaceous species (without excluding the possibility of Taxodiaceous affiliation) based in part on the observation of (minor) dehydroabiatic acid (IXa) by Py-GC-MS analysis. However, dehydroabietic acid is commonly found in many modern [11] and fossil resins from a wide range of families and hence is not of diagnostic value for chemotaxonomic purposes. The occurrence of callitrisate (X), however, is much more limited and hence is of greater chemotaxonomic interest.

Callitrisic acid (Xa) was first identified as a unique natural product isolated from the oleoresin of *Callitris columellaris *(Cupressaceae) by Carman and Deeth [22]. Subsequently, other authors have reported this product in resins and/or essential oils from *Callitris *[23], various *Juniperis *(Cupressaceae) *species *[23-30], and a number of non-conifers including *Salvia *[31], (although from the MS data reported by these authors it seems likely that the products observed are actually dehydroabiatic acid (IXa) and methyl dehydroabietate (IXb)), *Caleolaria *[32,33], *Rabdosia *[34], and the Rosaceae [35,36]. Callitrisate (X) has also been reported in thermally modified products derived from *Pinus *[37,38] but was not reported in analyses of unmodified resins from these species [39,40], so it is likely that these reports reflect secondary products rather than primary resin components. Other workers [11,41] have noted that reporting of compounds present in oleoresins from is often incomplete, but based on presently available data, it appears that occurrence of callitrisate (X) in modern conifer resins is limited to the Cupressaceae.

Correlations between macrofossil evidence and chemosystematics of co-deposited ambers need to be weighed with care. Ambers in any deposit will reflect the occurrence of resin producing species and copious resin producers may be disproportionately represented. Non resin producers or species with minor resin production will not be represented in the resin record, although these may dominate other palynological or paleontological indicators. For example, macrofossil evidence from the Fossil Forrest site (Canadian Arctic) indicates that the paleoenvironment at this site was dominated by *Metasequoia *and *Glyptostrobus *[42] but ambers from this site are dominated by *Pseudolarix *[14], which was apparently a minor species in that ecosystem [42].

Grimaldi et al [19] report that all of the ambers characterized in their study of Raritan ambers, including those found in unambiguous association with woody fossils are identical and are derived from a single botanical source, but for the reasons given earlier and in light of the data reported herein, this conclusion needs to be reassessed. Nevertheless, their observations and those of earlier workers (e.g. [43]) establish that at least some of the woody fossils found in these deposits are derived from resin producing species and that resins found in direct association with woody fossils are at least not grossly dissimilar to other ambers from these strata. Hence the characteristics of these woody fossils may be relevant to the chemotaxonomy of either or both of the amber forms identified in the present study.

Anatomical and (macro)structural studies of (resinous) fossil woods and cone scales associated with ambers from the Atlantic Coastal Plain are reviewed and summarized by Grimaldi et al [18,19]. In their study, Grimaldi [19] note that woody fossils collected in direct association with Raritan ambers possessed "crossfield pits [that] were generally in pairs and cupressoid-taxodioid in structure". Other (earlier) workers also recognized characteristics associated with the Cupressaceae in these fossils. (In fact, in the earliest report of investigation of the anatomical characteristics of these fossils [44] named the tree "*Cupressinoxylon bibbinsi*"). Resinous cone scales with characteristics similar to *Juniperus *also occur in association with these ambers (see [19] and works cited therein), thus strengthening the association of these fossils with the Cupressaceae. (Grimaldi et al [19] also cite unpublished data (M. Gandolfo, 1998) that fusinitized plant remains from the Raritan are "dominated by Cupressaceae".)

### Geochemistry

In addition to dehydroabietate (IX) and callitrisate (X), 16,17-dinor-dehydroabietate (XIV) and 16,17-dinor-callitrisate (XV) are also observed in the pyrolysates of these samples and in fact in some cases, these products predominate compared to the "parent" compounds (dehydroabietate and callitrisate).

**Figure 5:**
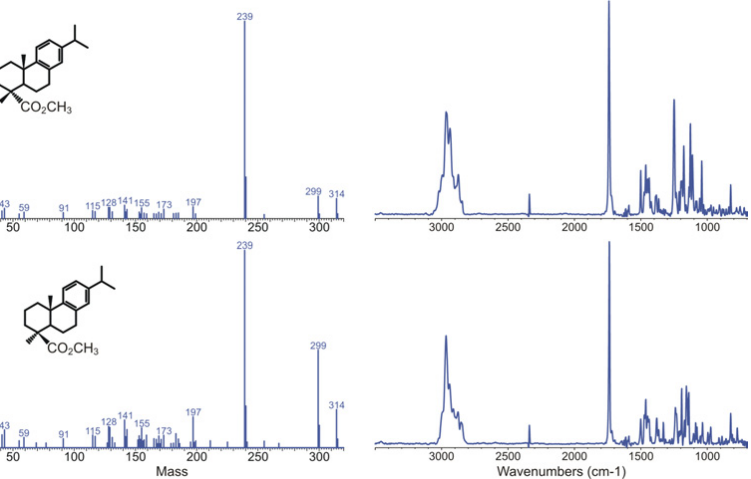
MS and matrix isolation FTIR data for methyl dehydroabietate (IXb) (upper) and methyl callitrisate (Xb) (lower).

Methyl dehydroabietate (IXb) and methyl callitrisate (Xb) can be differentiated on the basis of MS data alone, especially in samples where both are present, due to the enhanced relative intensity of the molecular ion and M-15 ions in callitrisate [45]. (Differentiation of these compounds is less certain in cases where only one epimer is present due to fact that the relative intensity of the M^+. ^and M-15^+ ^ions are in part determined by instrumental parameters.) However, confirmation of the A-ring stereochemistry is readily apparent from IR spectra, which exhibit strong and characteristic differences due to differences in the configuration of the A-ring carboxyl group (equatorial carbomethoxy → intense absorbance at ~1248 cm^-1^, axial carbomethoxy → moderately intense absorbance at ~1155 cm^-1^) [46,47] as illustrated in Figure 5. The same relationships hold true for nor-alkyl and dehydro analogs of these parent compounds and hence, using both MS and mi FTIR data it is possible to identify these products with a high degree of confidence.

**Figure 6:**
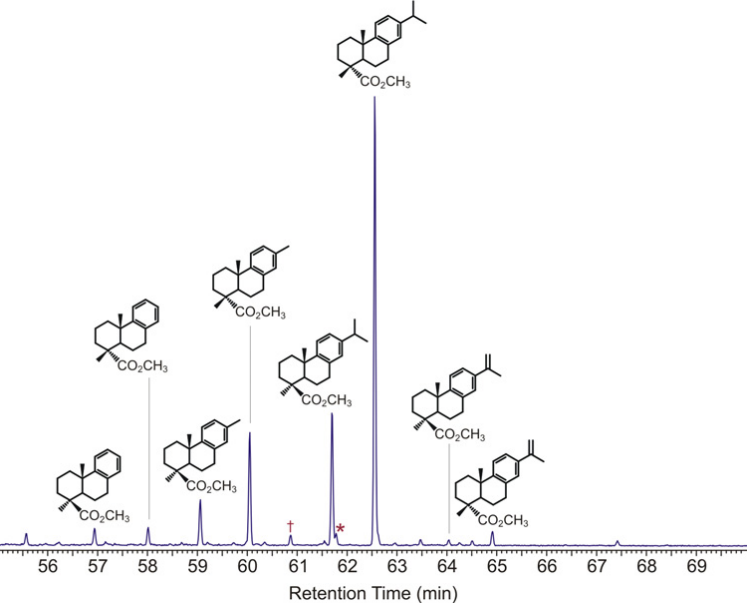
RIC (m/z = 239 + 237+ 225 + 211 + 197) for Raritan amber form 1 (Bleb 3) showing distribution of C-ring aromatic diterpenes. Compounds labeled † and * are assigned as methyl 17-nor dehydroabietate (XVIb) and methyl 17-nor calitrisate (XVIIb) respectively. However, due to the low abundance and coeleution of these eluants with other analytes, these assignments are tentative only.

**Figure 7:**
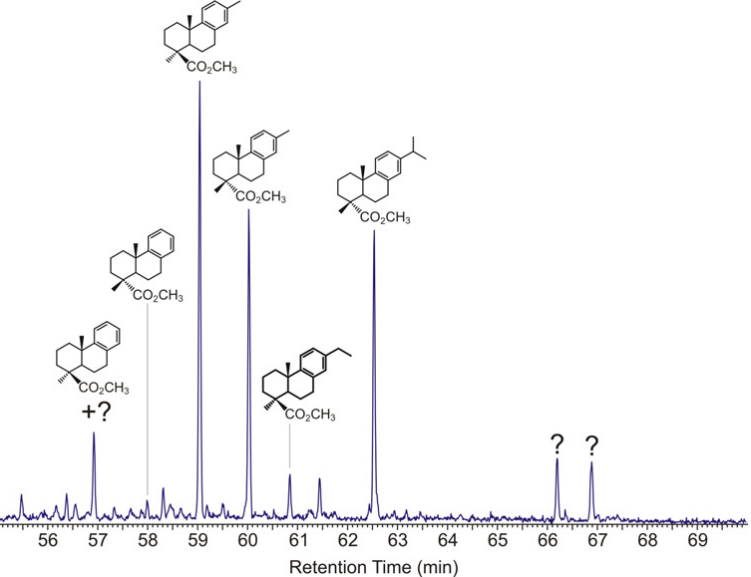
RIC (m/z = 239 + 237+ 225 + 211 + 197) Raritan Amber form 2 (Bleb 2) showing distribution of C-ring aromatic diterpenes.

To further investigate this, the distribution of various nor-methyl and dehydro analogues of dehydroabietate and callitrisate were examined. Reconstructed ion chromatograms (RIC) (m/z = 239 + 237+ 225 + 211 + 197) for samples of form 1 and 2 are illustrated in Figures 6 and 7, respectively. Supporting supplemental data are also given in Additional File 2. (Supplemental data are also available on line at http://suplimental data Figure 6, 7) These data indicate that in addition to 16,17-dinor dehydroabietate (XIV) and 16,17-dinor callitrisate (XV), the 17-nor (XVI and XVII) and 15,16,17-trinor (XVIII and XIX) analogues are also present. Abieta-8,11,13,15-tetraen-18-oic acid ("15–16 dehydro-dehydroabietate") (XX) and 15–16 dehydro-callitrisate (XXI) are also present as minor components in some samples. A-ring stereochemistry has been assigned based on analysis of both MS and FTIR data for individual analytes. (Note: due to low abundance and in some cases coelution with other eluants, identification of the 17-nor analogues (XVI and XVII) in some samples is based on the presence of characteristic ions and is, therefore, tentative).

The presence of the various analogues of both abietate and callitrisate suggests the possibility of a either a diagenetic degradation pathway from dehydroabietate and a parallel pathway from callitrisate, or a biological relationship emplaced during biosynthesis of the original resin. In this study, the availability of multiple samples collected from the same deposit (and therefore having identical geologic histories) and the existence of two distinct forms with differing compositions, allows definitive differentiation of these possibilities. If the relationship between these compounds were derived from a diagenetic (post depositional) degradation, then it can be assumed that alteration pathway will be the same in all samples. Hence for any given initial composition, the relative ratios of the parent compounds and their daughter products should be comparable in all cases. This is not observed. From the data illustrated in Figure 6, it would appear that in this sample, the relative ratios of the parent compounds and their "daughter" products are comparable. However, this relationship is not observed in other samples. The data illustrated in Figure 7 and in additional data for the remaining samples show wide variations in the ratios of all products.

This result establishes two things: Firstly, it establishes that diagenetic pathway(s) for this group of compounds need to be interpreted with care because of the potential for superimposition of biological signatures over potential diagenetic modifications. Secondly, it establishes that the distributions of compounds observed in these samples reflects the original resin composition and is, therefore, useful for chemotaxonomic assessment.

## Conclusion

Pyrolysis-GC-MS analysis of samples of amber collected from the Raritan Formation, New Jersey, clearly indicate the existence of two related-but-distinct forms. Both forms are Class Ib ambers. However, differences in the distributions of other diterpenoids unambiguously differentiate these materials and establish that ambers from this formation are derived from multiple related-but-distinct paleobotanical sources. The presence of both dehydroabietate (IX) and callitrisate (X) in all samples suggests that both forms are derived from species belonging to the Cupressaceae, and this conclusion is supported by the observation of cuparene (VIII) in form 1.

In addition to dehydroabietate (IX) and callitrisate (X), these ambers also contain significant amounts of related nor-, dinor- and trinor- analogues of these "parent" compounds. The distributions of these analogues, however, are not consistent with diagenetic alteration, suggesting that these products are biologically emplaced.

**Appendix 1:**
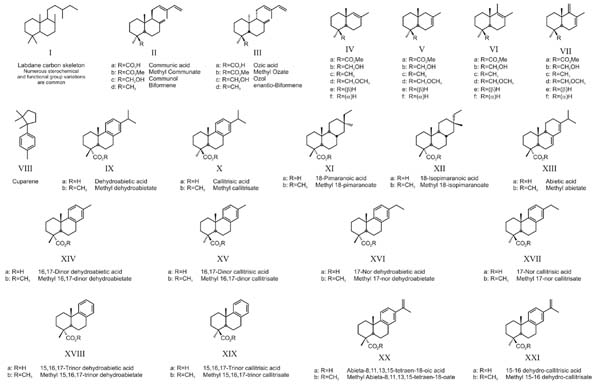
Structure key for structures referred to in the text.

## Supplementary Material

Additional File 1 Supporting interactive supplemental data for Figure 4, including machine readable structure and MS data are given in Additional File 1.zip. To access these data, download this file and unzip the compressed archive, ensuring that the embedded directory structure is preserved. Once uncompressed, simply open Figure 4.html. Javascript must be enabled in your web browser in order to fully access these files. These files will also be available on line via the Geochemical Transactions web site in the near future. Click here for file

Additional File 2 Supporting interactive supplemental data for Figure 6 and 7, including machine readable structure and MS data are given in Additional File 2.zip. To access these data, download this file and unzip the compressed archive, ensuring that the embedded directory structure is preserved. Once uncompressed, simply open Figure 6-7.html. Javascript must be enabled in your web browser in order to fully access these files. These files will also be available on line via the Geochemical Transactions web site in the near future. Click here for file
