# Supplementary material for: The nature and fate of natural resins in the geosphere. XII. Investigation of C-ring aromatic diterpenoids in Raritan amber by pyrolysis-GC-matrix isolation FTIR-MS
Source: Geochem Trans. 2006 Mar 1;7:2. doi: 10.1186/1467-4866-7-2 (PMC1459126; doi:10.1186/1467-4866-7-2)
Supplement: Additional File 2 — Supporting interactive supplemental data for Figure 6 and 7, including machine readable structure and MS data are given in Additional File 2.zip. To access these data, download this file and unzip the compressed archive, ensuring that the embedded directory structure is preserved. Once uncompressed, simply open Figure 6-7.html. Javascript must be enabled in your web browser in order to fully access these files. These files will also be available on line via the Geochemical Transactions web site in the near future. [file 1467-4866-7-2-S2.zip › Figure 6-Suplimental Data/Figure 6-7.htm]

Raritan Amber C-ring Aromatic Diterpenes


|  |  |
| --- | --- |
| Bleb #3 [Form 1] Bleb #5 [Form 1] Bleb #1 [Form 2] Bleb #2 [Form 2] Bleb #4 [Form 2]  Bleb #3 [Form 1] Bleb #5 [Form 1] Bleb #1 [Form 2] Bleb #2 [Form 2] Bleb #4 [Form 2] | |
| Interactrive GC-MS data set for Figures 6 and 7  Multi Ion Chromatogram (m/z=239+237+225+211+197) illustrating variation in the distribution of C-ring aromatic diterpenoids in Raritan Amber | Instructions |
